# Clustering of behavioral risk factors for chronic noncommunicable diseases in climacteric women

**DOI:** 10.31744/einstein_journal/2022AO6153

**Published:** 2022-02-02

**Authors:** Roberto Rodrigues Leite, Antônio Prates Caldeira, Josiane Santos Brant Rocha, Luiza Augusta Rosa Rossi-Barbosa

**Affiliations:** 1 Universidade Estadual de Montes Claros Montes Claros MG Brazil Universidade Estadual de Montes Claros, Montes Claros, MG, Brazil.

**Keywords:** Risk factors, Noncommunicable diseases, Climacteric, Women, Women’s health, Health behavior, Disease prevention, Obesity

## Abstract

**Objective:**

To estimate the prevalence of clustering of behavioral risk factors for chronic non-communicable diseases, as well as the associated factors in climacteric women.

**Methods:**

This is a cross-sectional, analytical study, with random selection of climacteric women, aged between 40 and 65 years, and registered in Family Health Strategy units. The dependent variable was clustering of three or more behavioral risk factors for chronic non-communicable diseases. The definition of associated variables was made after Poisson multiple regression analysis with robust variance.

**Results:**

We evaluated 810 women, and 259 (32.0%) had a clustering of risk factors. The main risk behaviors were physical inactivity and low fruit consumption. The variables associated with clustering of behavioral factors were age group 52-65-years, marital status without a partner, overweight/obesity, moderate to severe anxiety and depression symptoms.

**Conclusion:**

There was a considerable prevalence of women with three or more behavioral risk factors for chronic non-communicable diseases. Demographic variables and those related to health conditions were shown to be associated. Considering the results recorded, health services must provide differentiated care policies to climacteric women, seeking to alleviate high morbidity and mortality of chronic non-communicable diseases.

## INTRODUCTION

Chronic non-communicable diseases (NCD) are multifactorial, of non-infectious origin, long lasting, and develop over the course of life. They can generate functional disabilities and significant impairments, and are responsible for more than 60% of deaths worldwide.^(1)^ They affect the low-income population more intensely and account for considerable health care expenditure.^(2,3)^ They are characterized by long latency periods and are influenced by multiple risk factors.^(4,5)^

Chronic NCD are a major public health problem worldwide, and account for 46.0% of global burden of diseases affecting the world population. For this reason, they are considered one of the greatest challenges for health managers.^(1)^In Brazil, deaths from these causes correspond to twice the number of infectious diseases and highlight chronic NCD as the main public health problem.^(4,6,7)^Studies have shown an increase in cardiovascular diseases in people aged under 65 years and mainly women, with an increased number of hospitalizations.^(8,9)^

Physical inactivity, inadequate eating habits, obesity, alcohol, and soft drink abuse, in addition to smoking, are examples of behavioral (and thus modifiable) risk factors for the onset of chronic NCD.^(5,8,10)^ A cross-sectional study in 27 countries showed that most patients with coronary heart disease have unhealthy lifestyles, considering smoking, diet, and sedentary behavior, negatively affecting the control of major risk factors for chronic NCD, such as hypertension, elevated low-density lipoprotein cholesterol (LDL-c), and diabetes.^(11)^ Although the role of each of these risk factors has been well established individually, their clustering occurs repeatedly, and some have synergistic action; this simultaneous exposure is a reason for worsening and poor prognosis.^(12-14)^

There is little information on clustering of risk factors in the literature and even less addressing the group of climacteric women. It is believed that a better understanding of clustering of inappropriate behaviors in this population should enable implementing public policies, which are more effective to reduce injuries and deaths due to chronic NCD.

## OBJECTIVE

To estimate the prevalence of clustering of behavioral risk factors for chronic non-communicable diseases and associated variables among climacteric women.

## METHODS

This is a cross-sectional, analytical, population-based study, developed by the *Universidade Estadual de Montes Claros* (UNIMONTES), together with the Family Health Strategy teams of Montes Claros, in northern state of Minas Gerais.

The target population was composed of all climacteric women registered at 73 Family Health Strategy units of the municipality of the study, between August 2014 and August 2016. The sampling process was probabilistic, in two stages. In the first stage, a simple drawing of the Family Health Strategy units that would participate in the study was performed. In the second stage, the climacteric women registered in each team were identified and selected for the study by an equally random selection, respecting the proportionality of stratification according to the climacteric period (pre-, peri-, and postmenopausal). Premenopause begins at 40 years of age, lasting as long as the menstrual pattern remains similar to that of menacme. Perimenopause begins with menstrual irregularity (a decrease or increase in the interval between menstruations) and lasts up to 12 months after menopause. Finally, post-menopause comprises the period starting after the 12-month interval after menopause until the age of 65 years.^(15)^

The sample size was calculated considering a confidence level of 95.0%, margin of error of 5.0%, and estimated prevalence of 50.0% for the event studied, due to the lack of similar studies, and to have a larger sample. According to the methodology recorded in a previous study,^(16)^the sample was duplicated (Deff = 2), estimated to collect data from at least 760 women.

The inclusion criterion was to be registered in Primary Health Care services. Pregnant, puerperal, and bedridden women were excluded from the study. After the draw, the service users were invited to participate in the study through orientation and clarification about the proposal. All women who agreed to participate in the study were asked to sign the Informed Consent Form (ICF). On a previously scheduled day, the participants went to the Primary Health Care Unit (UBS - *Unidade Básica de Saúde*) for filling in the questionnaire, clinical assessment, and anthropometric measurements.

The data collection team was specially trained to standardize the interviews and anthropometric measurements. A pilot study was conducted to calibrate and evaluate the interviewers’ practice, and to measure the level of understanding of the questions. The women who participated in the pilot study were not included in the final study.

The response variable in this study was clustering of behavioral risk factors for chronic NCD, which were defined as the concomitance of three or more of the following factors: habitual consumption of meat with fat, habitual consumption of chicken with skin, excessive salt consumption, and low fruit consumption, regular consumption of soft drinks, smoking, and abusive consumption of alcoholic beverages. These items were researched based on the Surveillance of Risk and Protective Factors for Chronic Diseases by Telephone Survey (VIGITEL)^(17)^ and physical inactivity.^(18)^

Questioning about the consumption of meat with fat and chicken with skin contained five possible answers: always remove the visible excess; sometimes remove the visible excess; eat with fat or skin; do not eat red meat with a lot of fat or chicken with skin; do not eat red meat or chicken with skin. For the present study, these data were dichotomized, with responses two and three considered fatty meat consumption.

About excessive salt consumption, the possible answers were I never add salt to my plate of food; I almost always add salt, even without tasting it; I taste it and add salt if it lacks salt. Answer number two referred to excessive salt consumption, and the other answers to habitual, not abusive, salt consumption.

Alcohol abuse was considered for those women who reported having had four or more doses of alcoholic beverages on a single occasion in the past 30 days. A dose of alcohol was defined as the equivalent of one can of beer, one glass of wine, or one measured dose of hard liquor.^(17)^ Smoking was considered for all women who reported regular use of cigarettes or similar.

Habitual soft drink consumption was considered for women who reported consuming soft drinks three or more times a week. As for fruit consumption, low consumption was considered for those women who reported eating less than three portions of fruit per day.

Physical activity was investigated by the International Physical Activity Questionnaire (IPAQ), in its short version, already validated for Brazil.^(18)^ Physical inactivity was presumed for women who, according to the classification of the instrument, were identified as sedentary or insufficiently active, *i.e*., those who did not meet sufficient criteria as to frequency or duration. To consider a person as active, she should perform vigorous physical activity three or more days a week for at least 20 minutes, or moderate activity for 30 minutes more than 5 days a week, or 150 minutes of walking more than 5 days a week.^(18)^

The independent variables were assessed as follows: age range (40 to 45, 46 to 51, and 52 to 65 years), self-reported skin color (white, brown, black, and other), marital status (married, single, or separated/divorced/widowed), education (elementary, middle, and higher), salary (up to one and more than one minimum wage), climacteric stage (pre-, peri- and postmenopausal), intensity of climacteric symptoms (mild, moderate, and severe), body mass index (BMI; adequate, overweight, and obesity), depression (absent, mild, moderate, and severe), anxiety (minimal, mild, moderate, and severe), and quality of sleep (with and without impairment).

For the evaluation of climacteric symptoms, the Kupperman Menopausal Index was used, in which symptoms are graded as mild, moderate, and severe, through a structured questionnaire that evaluates vasomotor symptoms, paresthesia, insomnia, nervousness, sadness, weakness, arthralgia/myalgia, headache, palpitation, and tingling.^(19)^

As for emotional disorders, Beck’s anxiety and depression inventories were used, both validated in Brazil.^(20,21)^ Sleep quality was investigated using the Pittsburgh Sleep Quality Index (PSQI).^(22)^

Data were tabulated using the (SPSS). To analyze the association between clustering of risk factors for chronic NCD (dependent variable) and the independent variables, a bivariate analysis was performed using Pearson’s χ^2^ test. Those associated up to a level of 20.0% (p≤0.20) were selected for Poisson multiple regression analysis with robust variance. A significance level of 5.0% (p<0.05) was considered for the final model.

The research project was submitted to the Research Ethics Committee of the *Faculdades Integradas Pitágoras de Montes Carlos* where the investigation was conducted and approved under opinion Number 817.166 (CAAE: 36495714.0.0000.5109).

## RESULTS

We evaluated 810 women in the climacteric period. Of these, 259 (32.0%) had at least three risk factors. They were then considered as carriers of clustering of behavioral risk factors for chronic NCD.

Table 1 shows the individual prevalence of each of the risk factors evaluated that comprise the dependent variable. The most frequent factor in the study population was physical inactivity, while salt intake was the least common of all the others.

**Table 1:** Behavioral risk factors for chronic noncommunicable diseases among climacteric women seen by family health teams

Behavioral factors	n (%)
Physical inactivity	706 (87.2)
Low fruit consumption	522 (64.5)
Fat consumption in meat	132 (16.5)
Smoking	105 (13.1)
Abusive consumption of soft drink	92 (11.4)
Consumption of chicken skin	71 (8.9)
Consumption of alcohol	59 (7.3)
Excessive salt in food	15 (1.9)

The characterization of the group of women evaluated is presented on table 2. The mean age was 50.9±6.9 years, and most were in the age group 52 to 65 years. Most of the group self-reported they were brown, married, and had only elementary school education.

**Table 2:** Sociodemographic and clinical characteristics of climacteric women assisted by family health teams

Variables	n (%)
Age range, years	
40-45	223 (27.5)
46-51	220 (27.2)
52-65	367 (45.3)
Skin color, self-referred	
White	149 (18.5)
Brown	511 (63.5)
Black	101 (12.5)
Other	44 (5.5)
Marital status	
Married	523 (64.7)
Single	75 (9.3)
Separated/divorced/widowed	210 (26.0)
Education level	
Higher	42 (5.2)
Middle school	226 (28.0)
Elementary	540 (66.8)
Salary, minimum monthly wage*	
≤1	350 (43.2)
>1	460 (56.8)
Climacteric stage	
Premenopause	222 (27.4)
Perimenopause	241 (29.8)
Post-menopause	347 (42.8)
BMI	
Adequate	208 (25.7)
Overweight	311 (38.5)
Obesity	290 (35.8)
Intensity of climacteric symptoms	
Mild	494 (61.1)
Moderate	231 (28.6)
Severe	84 (10.3)
Depression	
Absent	487 (60.4)
Mild	207 (25.7)
Moderate	102 (12.7)
Severe	10 (1.2)
Anxiety	
Minimal	338 (41.9)
Mild	217 (26.9)
Moderate	156 (19.4)
Severe	95 (11.8)
Quality of sleep	
No impairment	255 (33.2)
Compromised	512 (66.8)

* The current value of the monthly minimum wage in 2014 was R$ 724,00.

BMI: body mass index.

Table 3 presents the results of the bivariate analysis between the characteristics of the evaluated group and clustering of behavioral risk factors for chronic NCD. The variables that were associated with clustering of three or more behavioral risk factors for chronic NCD were age, marital status, BMI, anxiety, and depression.

**Table 3:** Bivariate analysis between factors associated with clustering of behavioral risk factors for chronic noncommunicable diseases among climacteric women seen by family health teams

Variables	Clustering of risk factors		p value*

	≤2 factors	≥3 factors	

	n (%)	n (%)	
Age range, years			0.004
40-45	139 (62.9)	82 (37.1)	
46-51	138 (62.7)	82 (37.3)	
52-65	267 (73.8)	95 (26.2)	
Skin color, self-referred			0.041
White	89 (59.7)	60 (40.3)	
Nonwhite	444 (67.7)	212 (32.3)	
Marital status			0.008
With a partner	368 (71.0)	150 (29.0)	
Without a partner	175 (61.8)	108 (38.2)	
Education level			0.363
Higher/Middle school	176 (65.7)	92 (34.3)	
Elementary	367 (68.9)	166 (31.1)	
Salary, minimum monthly wage^†^			0.329
≤1	228 (65.9)	118 (34.1)	
>1	316 (69.1)	141 (30.9)	
Climacteric stage			0.037
Premenopause	138 (62.4)	83 (37.6)	
Perimenopause	159 (66.0)	82 (34.0)	
Post-menopause	247 (72.4)	94 (27.6)	
BMI			0.061
Adequate	170 (72.6)	64 (27.4)	
Overweight/obesity	374 (65.8)	194 (34.2)	
Intensity of climacteric symptoms			0.005
Mild	347 (70.8)	143 (29.2)	
Moderate	152 (66.4)	77 (33.6)	
Severe	44 (53.0)	39 (47.0)	
Depression			<0.001
Absent/mild symptoms	483 (70.2)	205 (29.8)	
Moderate/severe symptoms	59 (52.7)	53 (47.3)	
Anxiety			<0.001
Minimal/mild symptoms	398 (72.5)	151 (27.5)	
Moderate/severe symptoms	145 (57.8)	106 (42.2)	
Quality of sleep			0.031
No impairment	186 (73.5)	67 (26.5)	
Compromised	335 (65.8)	174 (34.2)	

* χ^2^ test; ^†^ prevailing value of the minimum wage in 2014: R$ 724,00.

BMI: body mass index.

The adjusted prevalence ratios with their respective confidence intervals are presented on table 4. The variables that were associated with clustering of three or more behavioral risk factors for chronic NCD were age group 52 to 65 years, marital status without a partner, overweight/obesity, moderate/severe anxiety symptoms, and moderate/severe depression symptoms.

**Table 4:** Variables associated with clustering of behavioral risk factors for chronic noncommunicable diseases climacteric women, seen by family health teams after multivariate analysis

Variables	Adjusted PR	95%CI	p value
Age range, years			
40-45	1.00		
46-51	1.01	0.79-1.29	0.916
52-65	1.50	1.18-1.91	0.001
Marital status			
With a partner	1.00		
Without a partner	1.34	1.10-1.64	0.005
BMI			
Adequate	1.00		
Overweight/obesity	1.27	1.01-1.61	0.047
Anxiety			
Minimal/mild symptoms	1.00		
Moderate/severe symptoms	1.39	1.12-1.72	0.003
Depression			
Absent/mild symptoms	1.00		
Moderate/severe symptoms	1.32	1.03-1.70	0.031

PR: prevalence ratio; 95%CI: 95% confidence interval; BMI: body mass index.

## DISCUSSION

The present study made it possible to estimate a considerable percentage of climacteric women with a clustering of behavioral risk factors for chronic NCD. A study conducted in southern Brazil with women aged between 18 and 90 years showed a prevalence of 17.1% of three or more risk factors. The clustering of risk factors, especially the modifiable ones, related to lifestyle, is an important public health issue, considering the synergistic action of these factors, which implies an increased mortality rate in middle-aged women.^(6,8,12)^ A recent Chinese study showed compromised cardiovascular health in women after increased clustering of two or more risk factors during the climacteric period.^(23)^

Among the seven risk factors considered, physical inactivity was the most prevalent. Similar results were observed in studies with a prevalence of 73.0%^(11)^ and 84.0%,^(22)^identified among women who did not meet the target of at least 30 minutes of physical activity five times a week. Another study, conducted in São Paulo with climacteric women, estimated 55.4% as sedentary or insufficiently active.^(24)^ Studies showed that this is the most important and prevalent risk factor among women.^(14,25,26)^ This denotes the importance of encouraging physical activity as a strategic pillar together with other lifestyle changes.

Studies indicate that programs focused on health promotion intervention and awareness on multiple risk factors, such as smoking, physical activity and diet simultaneously, are more effective in reducing chronic NCD.^(13,14)^ It is believed that people with clustering of risk factors can be motivated and sensitized more quickly, so that they are more responsive to intervention programs.^(13)^

The low consumption of fruit, the second most prevalent factor, may be related to the high cost it represents, especially when considering low-income populations, or even because they are being neglected, given the wide availability of fast foods and processed products.^(27)^ A study evaluating markers of food consumption found, with the greater purchasing power through the *Bolsa Família* Program, there was an increase in the consumption of foods considered unhealthy.^(28)^ It is still noteworthy that unhealthy eating patterns encompass several other deleterious behaviors, such as eating meat with excess fat, which are proportional to the low consumption of fruits.^(29)^ The consumption of meat with visible fat shows a relation with the population’s misinformation or low incentive for a healthier diet, since the removal of excess fat could easily be performed before preparing or eating the meat.^(30)^

The variables that, after multiple analysis, were shown to be associated with clustering of three or more modifiable risk factors for chronic NCD were higher age among climacteric women (equal to or greater than 52 years), living without a partner, high BMI, presence of mild to severe symptoms of anxiety, and presence of moderate to severe symptoms of depression.

Regarding age, in a study carried out in Paraná with the general population, a high association was observed in the elderly population with clustering of two or more risk factors. Since age is a non-modifiable factor, a substantial increase in the risk of ischemic heart disease is observed when in the presence of clustering of other factors.^(25)^

Regarding marital status, a study demonstrated women living without a partner are more susceptible to unhealthy lifestyle habits, which can be explained by the lack of a stable emotional condition or family support.^(26)^

The association between clustering of risk factors with high BMI was already expected, since physical inactivity and poor dietary habits will inexorably culminate in weight gain.^(26)^ Obesity in the general population is more prevalent in women, and those with visceral obesity have a higher prevalence of subclinical heart disease. In the same study, susceptibility to depression due to obesity was reported.^(31)^

No studies were found addressing the relation of clustering of risk factors or with similar design with the variables anxiety and depression. However, it is observed that depression in women is associated with cardiac events.^(32)^ A research showed an association between clustering of psychosocial factors and risk factors for cardiovascular diseases in African-American women.^(33)^ It is possible to infer that women with these psychiatric disorders are less motivated to self-care with health and, therefore, more likely to have more risk factors. In addition, a high prevalence of anxiety and depression is perceived in climacteric women, most likely related to hormone changes and disorders related to this phase of life.^(34,35)^

Awareness campaigns, through the media and other vehicles, about the risk of cardiovascular disease in women should be fostered.^(9)^ Updated guidelines specific to the prevention of cardiovascular disease in women are desirable to aid clinical decisions.^(32)^

The results should be considered under some limitations. Some comparisons were made with population groups different from this study - climacteric women. This is justified by the fact that data on clustering of risk factors in this population are scarce in the literature. The generalization of data is restricted to the population of women assisted by the Family Health Strategy teams. It is also important to note that the study covers a single municipality in a poor region, and that cities in other regions may have different characteristics. It should be noteworthy that women assisted in the private network or in other levels of complexity of care were not included in the study.

## CONCLUSION

The relevance of the results observed in this research should be highlighted, since they allowed us to verify the prevalence of clustering of behavioral risk factors for chronic noncommunicable diseases and the associated variables among climacteric women. These data are scarce in the literature so far, both in general and specific populations. It is also evident the study had a representative sample and used instruments validated in the literature.

Therefore, this study can contribute to a better understanding of the theme, as well as enable the formulation and implementation of policies aimed to address these factors together, and thus reduce morbidity and mortality associated with chronic health problems.
